# The survival benefit of different lymph node yields in radical prostatectomy for pN1M0 prostate cancer patients: Implications from a population-based study

**DOI:** 10.3389/fonc.2022.953069

**Published:** 2022-08-11

**Authors:** Jieping Hu, Yue Yu, Wei Liu, Jialei Zhong, Xiaochen Zhou, Haibo Xi

**Affiliations:** Department of Urology, The First Affiliated Hospital of Nanchang University, Nanchang City, China

**Keywords:** radical prostatectomy, lymph node dissection, Gleason score, tumor stage, prognosis (carcinoma)

## Abstract

**Background and objectives:**

The extent and survival benefits of lymph node dissection (LND) in radical prostatectomy (RP) for pN1M0 prostate cancer (PCa) patients remained unclear and were controversial. This study aimed to determine the survival benefit of different lymph node yields in RP for pN1M0 PCa patients.

**Methods:**

pN1M0 PCa patients who received RP and LND were identified in Surveillance Epidemiology and End Results (SEER) (2010–2015). Patients were divided into two groups in SEER based on the removal of one to three regional lymph nodes (LND1 group) or four or more regional lymph nodes (LND4 group). Kaplan–Meier methods were used to calculate cancer-specific survival (CSS) and overall survival (OS).

**Results:**

In total, 2,200 patients were identified; 264 patients received LND1 and 1,936 patients received LND4. CSS had no significant difference between the LND4 and LND1 groups (101mon vs. 98mon, *p* = 0.064), and OS was higher in LND4 patients compared with LND1 patients (97mon vs. 93mon, *p* = 0.024); for patients with Gleason score = 9 or 10 and T3b or T4, 5-year OS was higher in patients undergoing LND4 (80.9%; 95% CI, 79.0–82.8) compared with those undergoing LND1 (67.5%; 95% CI, 60.8–74.2) (*p* = 0.009).

**Conclusion:**

More lymph node yield provided better survival for patients with Gleason score = 9 or 10 and T3b or T4, but not for other pN1M0 PCa patients. The extent of LND would be determined after a comprehensive evaluation including Gleason score, tumor stage, and the general condition of the patient.

## Introduction

Lymph node dissection (LND) is the gold standard for nodal staging in prostate cancer (PCa) patients undergoing radical prostatectomy (RP), and yields survival benefits compared to RP alone in a given population (1). D’ Amico risk, preoperative biopsy International Society of Urological Pathology (ISUP) grade, and risk of nodal metastases predicted by nomogram are applied to stratify patients to guide LND decisions (1–3). Previous studies have questioned the significance of LND, which may not have any direct benefit on cancer outcomes and may instead result in more complications (4). Only about 15% of PCa patients harbor lymph node invasion at RP and LND (5); these lymph node-positive patients who benefit from LND may not stratify by D’ Amico risk or ISUP grade. Based on state-of-the-art available tumor information, including multiparametric magnetic resonance imaging, novel nomograms predicting lymph node invasion was outstanding (6, 7), European Association of Urology (EAU) recommends that the risk of nodal metastases of >7% predicted by nomogram is an indication to perform extended LND (1), and the indications for limited LND or extended LND were not well characterized. Extended LND provided better pathological staging and survival (8, 9); on the other hand, the more extensive the LND, the greater the adverse outcomes in terms of postoperative complications (10, 11). A more rigorous assessment and selection criteria would be developed to help decide the extent of LND in RP for pN1M0 PCa patients according to survival benefit. Therefore, we aimed to stratify patients into groups and analyze survival using the Surveillance, Epidemiology, and End Results (SEER) program database.

## Methods

The SEER currently collects and publishes cancer incidence and survival data from population-based cancer registries covering approximately 48.0% of the U.S. population. It included incidence and population data associated with age, sex, race, year of diagnosis, and disease information. The SEER Program registries routinely collect data on patient demographics, primary tumor site, tumor morphology and stage at diagnosis, the first course of treatment, and follow-up for vital status (12). Within all 18 SEER-based registries, cases were identified as men diagnosed with adenocarcinoma of the prostate (Site and morphology. Behavior code, International Classification of Disease for Oncology [ICD-O-3] code 8140, Site and morphology. Primary site C61.9-prostate gland) between 2010 and 2015. Patients documented with American Joint Committee on Cancer (AJCC) Cancer Staging, Gleason score, prostate-specific antigen (PSA) value, one to three or four or more regional lymph nodes removed, and survival months were included. Tumor stage was confirmed by pathology, imaging methods such as conventional CT, bone scan, and PET/CT were used to diagnose distant metastasis.

Patients were divided into two groups: one to three regional lymph nodes (LND1), and four or more regional lymph nodes (LND4). Cases with unknown PSA value, Gleason score, and T stage were excluded. Age, race, PSA value, Gleason score, and T stage were analyzed for predicting overall and cancer-specific survival. Univariable logistic regression analyses assessed predictors of survival, nomograms predicting OS and CSS were developed for each group, and survival benefit was estimated when comparing the LND1 group and the LND4 group.

For categorical variables, the *χ* ^2^ test was used to evaluate the difference. COX multivariate survival analysis was to identify independent prognostic variables with *p* < 0.05. All statistical tests were performed using the R statistical package v.3.6.3 (The R Project for Statistical Computing, www.r-project.org). All tests were two-sided, and a *p*-value < 0.05 was considered statistically significant.

## Results

**Figure 1:**
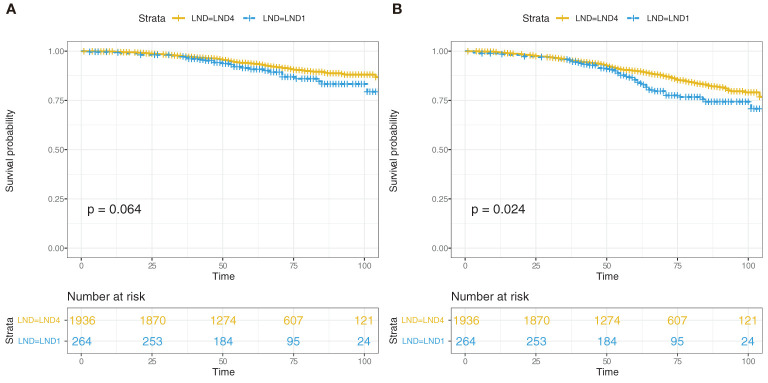
Kaplan–Meier estimated of cancer-specific survival (CSS) **(A)** and overall survival (OS) **(B)** in the pN1 patients who received radical prostatectomy and lymph node dissection according to one to three regional lymph nodes (LND1) or four or more regional lymph nodes (LND4).

A total of 2,200 pN1 patients who underwent RP+LND were identified. Of those, 264 received LND1 vs. 1,936 who received LND4. With a median follow-up of 60 months (interquartile range [IQR]: 45–81), 166 patients (7.5%) died of PCa: 29 (17.5%) in the LND1 group, and 137 (82.5%) in the LND4 group; all-cause mortality was 18.2% for the LND1 group compared to 11.9% for the LND4 group (*p* = 0.004). Patient characteristics are listed in **Table 1**. CSS was not significantly different between the LND4 and LND1 group (101mon vs. 98mon, *p* = 0.064); however, OS was higher in LND4 patients compared with LND1 patients (97mon vs. 93mon, *p* = 0.024) (**Figure 1**).

**Table 1:** Characteristics of included patients.

Characteristic		One to three regional lymph nodes (*n* = 264)	Four or more regional lymph nodes (*n* = 1,936)	*p*-value
Age (years, mean ± SD)		61.5 ± 6.8	62.2 ± 7.1	0.42
Race	Asian or Pacific Islander	10	95	<0.001
	Black	63	272	
	White	191	1569	
PSA (ng/ml)		15.10 ± 14.82	16.32 ± 16.54	0.048
	<10	124	932	0.086
	10–20	90	548	
	>20	50	456	
Gleason score	5	1	0	<0.001
	6	3	12	
	7	144	874	
	8	21	280	
	9	90	755	
	10	5	15	
T stage	T1c	0	4	0.419
	T2a	5	17	
	T2b	3	23	
	T2c	39	230	
	T3a	75	526	
	T3b	110	907	
	T4	32	229	
Risk	Low	1	2	0.284
	Medium	0	9	
	High	263	1925	

To determine the optimal extent of LND in RP for pN1M0 PCa patients, Cox regression analysis was performed based on the extent of LND, age, race, Gleason score, PSA value, and T stage. Gleason score, PSA value, and T stage were significantly associated with survival (**Supplementary Table 1**). CSS and OS predominantly correlate with Gleason score [Exp(B) = 2.65 and 1.78 for CSS and OS, respectively, **Supplementary Table 1**], and subgroup analysis was performed according to Gleason score. The goal of the subgroup was to find the survival benefit of LND4 compared with LND1 within the group. For patients with Gleason score = 9 or 10, OS had no significant difference between LND4 and LND1 when patients had a stage ≤T3a. For 227 patients with Gleason score = 9 or 10 and T stage < T3b, 5-year OS was not different between LND4 (88.1%; 95% CI, 85.7–90.5) and LND1 (85.0%; 95% CI, 78.1–91.9) (*p* = 0.602, **Supplementary Figure 1A**). For 638 patients with Gleason score = 9 or 10 and T3b or T4, 5-year OS was higher in patients undergoing LND4 (80.9%; 95% CI, 79.0–82.8) compared with LND1 (67.5%; 95% CI, 60.8–74.2) (*p* = 0.009, **Supplementary Figure 1B**). For 301 patients with Gleason score = 8, Cox regression analysis did not find any association between OS and extent of LND, age, race, PSA value, or T stage. For 1,018 patients with Gleason score = 7, race was associated with OS; patients were divided into Asian or Pacific Islander (42 cases), Black (195 cases), and White population (781 cases), and T stage was associated with OS in the White population. Further analysis indicated that patients with T3a, T3b, and T4 stage who received LND4 were found to have preferred OS compared to those who received LND1 (**Supplementary Figures 2A, B**). Only 16 patients had Gleason score ≤6, and subgroup analysis was not performed.

**Figure 2:**
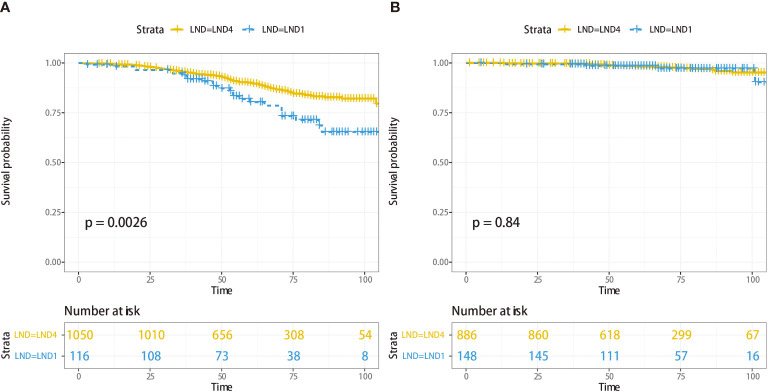
Cancer-specific survival analysis for 1,166 patients with Gleason score ≥8 according to LND1 or LND4 **(A)**, and 1,034 patients with Gleason score ≤7 according to LND1 or LND4 **(B)**.

Cox regression analysis indicated that Gleason score was also an important predictor for CSS; Gleason score ≥8 was the cutoff for LND4 after step-by-step analysis. Kaplan–Meier analysis of 1,166 patients with Gleason score ≥8 suggested that 5-year CSS was 80.3% (95% CI, 76.0–84.7) for LND1 and 89.9% (95% CI, 88.8–91.0) for LND4 (*p* = 0.003, **Figure 2A**), whereas 5-year CSS was not significantly different between LND4 and LND1 (98.2%; 95% CI, 97.7–98.7 vs. 98.5%; 95% CI, 97.5–99.5) (*p* = 0.844) in 1,034 patients with Gleason score ≤7 (**Figure 2B**). It was noted that patients with Gleason score ≥8 and T stage ≤T3a had comparable CSS between the LND4 and LND1 groups (*p* = 0.928).

## Discussion

Pathological N1M0 PCa was a multifaceted disease; age, race, marital status, positive surgical margins, pathological stage, positive nodes number, PSA level, and pathological Gleason score were prognostic factors for patients who received RP (5, 13, 14). Few studies focused on the oncological outcomes according to the number of LND. A recent prospective, single-center phase 3 trial confirmed that extended LND provided better pathological staging, while differences in early oncological outcomes were not demonstrated (8). The therapeutic role of extended LND to remove occult micrometastases in PCa patients undergoing RP probably existed (9); due to the low incidence of lymph node metastases in RP, populations in the studies were relatively small. Moreover, EAU risk stratification based on D’Amico stratification was commonly applied for stratifying patients who received extended LND or limited LND (15, 16); this stratification system was mainly predicting biochemical recurrence, and its prognostic utility may be weak. It was obvious that the extent of LND was meaningless for lymph node-negative patients; LND aimed to eliminate the cancerous lymph node. Thankfully, nomograms and new techniques were developed for predicting lymph node metastases (2, 7, 17, 18), and the extent of LND was worthy of in-depth research. However, so far, the extent of LND during RP for the management of pN1M0 PCa remained unclear, and novel risk categories for deciding the extent of LND were desperately needed.

In our study, a total number of 2,200 pN1M0 PCa patients were included. LND was divided into two groups: those with one to three regional lymph nodes and those with four or more regional lymph nodes; pN1M0 patients who received more lymph node yield obtained a better overall survival compared with those who received less LND, whereas cancer-specific survival was not different between the two groups; subgroup analysis indicated that patients with Gleason score >8 and T stage >T3a, or white race with Gleason score = 7 and T3a, T3b, T4 stage were the population who would benefit from LND4 in terms of OS. CSS was improved for patients with Gleason score >7 and T stage >T3a who received LND4 when compared with those who received LND1. These findings prompted new indications for LND4, and the number of LNDs that contributed to survival may depend on Gleason score and T stage in pN1M0 patients.

Recently, a single-center randomized trial including 1,440 clinically localized PCa patients addressed the therapeutic benefit of extended LND versus limited LND. Patients who received extended LND did not have lower biochemical recurrence rates compared to those who received limited LND within a median follow-up of 3.1 years (hazard ratio 1.04, 95% CI 0.93–1.15; *p* = 0.5) (19). Only 12% (81/700) of LLND and 14% (100/740) of extended LND patients suffered from lymph node metastases, and most patients who were lymph node negative and at the T1c stage may contribute to the outcome. Another study retrospectively analyzed data from 378 patients who underwent robot-assisted RP with LND. Patients were classified into three biochemical recurrence risk groups; therapeutic benefit may likely occur when extended LND was performed for patients with localized PCa at intermediate risk or higher (20). It provided a clue that risk stratification may help to decide the extent and number of LND, and we speculated that LND may be less likely to promote prognosis for pN0 PCa patients, and that LND was important for pN1 patients and the extent would depend on multiple factors.

The definitions of extended LND and limited LND were diverse in literature. Extended LND included the obturator, external iliac, internal iliac, common iliac, and presacral regions (nine fields) bilaterally, and limited LND was limited to the obturator region bilaterally (two fields) (8), whereas extended LND may include removal of the external iliac, hypogastric, and obturator fossa nodal groups, and limited LND may include surgical removal of the nodal packet under the external iliac vein and above the obturator nerve (19). Our study brought a new proposal about the number of LND based on SEER datasets, the number of lymph node yield was calculated, survival benefit was compared between groups, and LND4 improved survival for subgroup patients but not all pN1 PCa patients. Heterogeneity may exist among pN1 patients. A previous study analyzed the pathological information of 427 patients who received RP; a median of 16 lymph nodes were removed, a total of 35 (8.2%) had lymph node metastases, and of those patients, 24 (69%) had positive lymph nodes in only one of the three areas, namely, the external iliac in 4 (11%), the obturator in 9 (26%), and the hypogastric in 11 (31%) patients; 80% lymph node metastases patients had only one (49%) or two (31%) positive nodes (21). This information suggested that some regional nodes may be insignificant and could be safely excluded from the LND (22). Hypogastric and obturator fossa nodal might be removed for all pN1 patients because of their high incidence of metastases. It was critical to identify the positive lymph node before or during surgery; approximately 7% of lymph node metastases were detected outside the conventional extended LND template (23). We considered the extent of LND evaluated by risk stratification (mainly Gleason score and T stage in our study) outweighed an ELND template for pN1 PCa patients.

Imaging results such as CT/PET-CT and multiparametric MRI/PET-MRI had low sensitivity in identifying lymph node metastases (24, 25). Nomograms had been built for predicting lymph node metastases (2, 6); these tools also mainly relied on Gleason score and T stage, and we found that the two factors had an impact on the survival benefit of LND, and that extended LND was more likely to remove occult lymph node metastases, as higher Gleason score and T stage were risk factors for lymph node metastasis (26–28). We would like to point out that this study found Gleason score >8 and T stage >T3a was an indication for more lymph node yield.

Several biases were impossible to eliminate in this study, as it was a retrospective analysis based on SEER datasets. Some information was not available, such as treatment in the perioperative period, although adjuvant androgen deprivation therapy and adjuvant radiotherapy alone had no cancer-specific survival or overall survival advantages over observation. Adjuvant androgen deprivation therapy plus adjuvant radiotherapy yielded a survival benefit compared to observation and adjuvant androgen deprivation therapy (5), and distinct treatment before or after surgery could influence the survival analysis of LND. Positive surgical margin status was not recorded; a positive surgical margin length of > 6.0 mm (*p* = 0.003) was a significant predictor of biochemical recurrence (29). Another factor that was not reported but influences survival was the number of positive lymph nodes; a higher number of positive lymph nodes resulted in a poorer prognosis (5), three positive lymph nodes represent the best prognostic cutoff in node-positive PCa patients, and patients with one to three positive lymph nodes showed higher cancer-specific mortality-free survival estimates as compared with their counterparts with >3 metastatic lymph nodes (30). Last, because there are only two groups (less than or equal to three LNDs, or greater than four LNDs) in the SEER database, further grouping of lymph node number is limited, but this does not affect our conclusion. For some patients with Gleason score ≤8, T stage ≤T3a PCa, it may not be necessary to perform a conventional 12–16 LND; one to three suspected lymph nodes can be dissected for the pathological stage. PSA assessment early after RP+LND has an important prognostic role in the prediction of clinical recurrence and cancer-specific survival in node-positive patients. Postoperative adjuvant therapy including endocrine therapy may plays an important role in patients with lymph node metastasis. Risk stratification is recommended based on the PSA value at 6 weeks after surgery; patients with a complete biochemical response early after surgery share more favorable oncologic outcomes than those with PSA persistence (PSA ≥ 0.1 ng/ml), and a urologist would properly perform postoperative patient management according to PSA value (31).

## Conclusions

Our results suggested that the higher lymph node yield in RP conferred a survival advantage for pN1 PCa patients; subgroup analysis indicated that patients with Gleason score >8 and T stage >T3a were the main beneficiaries from LND4. The extent of LND would be determined after a comprehensive evaluation including Gleason score, tumor stage, and the general condition of the patient. Due to the inherent characteristic of SEER datasets, only a number range of LND was recorded, and specific lymph node numbers and regions were not documented. We expect a large long-term follow-up prospective trial to compare the survival benefits of different numbers of LNDs in pN1 PCa.

## Data availability statement

The raw data supporting the conclusions of this article will be made available by the authors, without undue reservation.

## Ethics statement

Ethical review and approval was not required for the study on human participants in accordance with the local legislation and institutional requirements.

## Author contributions

JH: Study concept and design, acquisition of data, drafting of the manuscript, and statistical analysis. YY: Acquisition of data, analysis, and interpretation of data. WL: Acquisition of data, analysis, and interpretation of data. JZ: Drafting of the manuscript. XZ: Drafting of the manuscript. HX: Study concept and design, analysis, and interpretation of data, drafting of the manuscript. All authors contributed to the article and approved the submitted version.

## Funding

This study was supported by the Jiangxi Provincial Natural Science Foundation (No: 20202BAB216033).

## Conflict of interest

The authors declare that the research was conducted in the absence of any commercial or financial relationships that could be construed as a potential conflict of interest.

## Publisher’s note

All claims expressed in this article are solely those of the authors and do not necessarily represent those of their affiliated organizations, or those of the publisher, the editors and the reviewers. Any product that may be evaluated in this article, or claim that may be made by its manufacturer, is not guaranteed or endorsed by the publisher.

## Supplementary material

Supplementary Figure 1 Overall survival analysis for 227 patients with Gleason score = 9 or 10 and T stage <T3b received LND1 or LND4 **(A)**, or 638 patients with Gleason score = 9 or 10 and T3b or T4 received LND1 or LND4 **(B)**.

Supplementary Figure 2 Overall survival of 781 White populations with Gleason score = 7 according to LND1 or LND4 **(A)**, subgroup survival analysis for patients with T3a, T3b, and T4 stage was performed according to LND1 or LND4 **(B)**.
