# Supplementary material for: The survival benefit of different lymph node yields in radical prostatectomy for pN1M0 prostate cancer patients: Implications from a population-based study
Source: Front Oncol. 2022 Aug 11;12:953069. doi: 10.3389/fonc.2022.953069 (PMC9404339; doi:10.3389/fonc.2022.953069)
Supplement: Supplementary file 3 [file Table_1.docx]

| Supplement Table 1. Cox regression analysis for CSS and OS | | | | | | | |
| --- | --- | --- | --- | --- | --- | --- | --- |
| CSS | B | SE | Wald | P value | Exp(B) | 95.0% Exp(B) lower CI | 95.0% Exp(B) upper CI |
| Age | -0.021 | 0.011 | 3.875 | 0.049 | 0.979 | 0.959 | 1 |
| GleasonScore | 0.975 | 0.107 | 82.483 | 0 | 2.65 | 2.147 | 3.27 |
| PSA | 0.008 | 0.004 | 4.282 | 0.039 | 1.008 | 1 | 1.016 |
| LND | 0.488 | 0.205 | 5.656 | 0.017 | 1.629 | 1.09 | 2.435 |
| Race | -0.034 | 0.145 | 0.056 | 0.812 | 0.966 | 0.728 | 1.283 |
| T stage | 0.435 | 0.104 | 17.399 | 0 | 1.545 | 1.259 | 1.895 |
| OS | B | SE | Wald | P value | Exp(B) | 95.0% Exp(B) lower CI | 95.0% Exp(B) upper CI |
| Age | 0.009 | 0.008 | 1.069 | 0.301 | 1.009 | 0.992 | 1.025 |
| GleasonScore | 0.574 | 0.07 | 67.464 | 0 | 1.776 | 1.548 | 2.036 |
| PSA | 0.007 | 0.003 | 4.789 | 0.029 | 1.007 | 1.001 | 1.014 |
| LND | 0.467 | 0.159 | 8.598 | 0.003 | 1.595 | 1.167 | 2.179 |
| Race | -0.066 | 0.11 | 0.363 | 0.547 | 0.936 | 0.754 | 1.162 |
| T stage | 0.268 | 0.073 | 13.407 | 0 | 1.307 | 1.133 | 1.509 |
